# Preoperative chemoradiotherapy for locally advanced low rectal cancer using intensity-modulated radiotherapy to spare the intestines: a single-institutional pilot trial

**DOI:** 10.1093/jrr/rrab106

**Published:** 2021-11-23

**Authors:** Katsuyuki Sakanaka, Kota Fujii, Yuichi Ishida, Nobutaka Mukumoto, Koya Hida, Hiroyuki Inoo, Yoshiharu Sakai, Takashi Mizowaki

**Keywords:** rectal cancer, intensity-modulated radiotherapy (IMRT), preoperative chemoradiotherapy, acute adverse event

## Abstract

The irradiated volume of intestines is associated with gastrointestinal toxicity in preoperative chemoradiotherapy for rectal cancer. The current trial prospectively explored how much of the irradiated volume of intestines was reduced by intensity-modulated radiotherapy (IMRT) compared with 3-dimensional conformal radiotherapy (3DCRT) and whether IMRT might alleviate the acute gastrointestinal toxicity in this population. The treatment protocol encompassed preoperative chemoradiotherapy using IMRT plus surgery for patients with clinical T3–4, N0–2 low rectal cancer. IMRT delivered 45 Gy per 25 fractions for gross tumors, mesorectal and lateral lymph nodal regions, and tried to reduce the volume of intestines receiving 15 Gy (V_15 Gy_) < 120 cc and V_45 Gy_ ≤ 0 cc, respectively, while keeping target coverage. S-1 and irinotecan were concurrently administered. Acute gastrointestinal toxicity, rates of clinical downstaging, sphincter preservation, local regional control (LRC) and overall survival (OS) were evaluated. Twelve enrolled patients completed the chemoradiotherapy protocol. The volumes of intestines receiving medium to high doses were reduced by the current IMRT protocol compared to 3DCRT; however, the predefined constraint of V_15 Gy_ was met only in three patients. The rate of ≥ grade 2 gastrointestinal toxicity excluding anorectal symptoms was 17%. The rates of clinical downstaging, sphincter preservation, three-year LRC and OS were 75%, 92%, 92% and 92%, respectively. *In conclusion,* preoperative chemoradiotherapy using IMRT for this population might alleviate acute gastrointestinal toxicity, achieving high LRC and sphincter preservation; although further advancement is required to reduce the irradiated volume of intestines, especially those receiving low doses.

## INTRODUCTION

Preoperative chemoradiotherapy for locally advanced rectal cancer reduces the local recurrence while preserving anal functions and has been commonly used in Western countries. Similar to the trends in Western countries, it has become recommended by the Japanese Society for Cancer of the Colon and Rectum guidelines 2019 [1], for rectal cancer with a high risk of local recurrence. S-1 is an oral fluoropyrimidine anticancer agent combining tegafur, a prodrug of 5-fluorouracil, with gimeracil and oteracil potassium in a molar ratio of 1:0.4:1. Gimeracil is a dihydropyrimidine dehydrogenase inhibitor that acts to maintain high levels of 5-fluorouracil in plasma and has been suggested to demonstrate radiosensitizing activity [2]. The recommended dose-fraction of radiotherapy and dosage of concurrent S-1 plus irinotecan in preoperative chemoradiotherapy has been explored in a multi-institutional phase I study in Japan, for patients with rectal cancer [3]. The phase II trial showed a promising pathological complete response rate [4]. Preoperative chemoradiotherapy using S-1 and irinotecan is one of the promising regimens for rectal cancer.

One of the common adverse events in preoperative chemoradiotherapy for rectal cancer is acute gastrointestinal toxicity. Rates of more than grade 2 and grade 3 acute diarrhea were reported as 35% [5] and 7–13% [3, 6–8] respectively, in preoperative chemoradiotherapy for rectal cancer using 3-dimensional conformal radiotherapy (3DCRT) with fluorinated pyrimidine analogs plus irinotecan. Gastrointestinal toxicities are associated with irradiated volumes of intestines [9]. Intensity-modulated radiotherapy (IMRT) suppressed these and is expected to decrease gastrointestinal toxicities; however, the clinical usefulness of IMRT in preoperative chemoradiotherapy for rectal cancer has not yet been established. As of October 2016, phase II trials of IMRT mainly focused on its effectiveness for rectal cancer [10–14]. One multi-institutional phase II study only focused on the reduction of acute gastrointestinal toxicities by IMRT and failed to show it [15]. One of the possible reasons for the failure of IMRT to reduce acute gastrointestinal toxicity might be that the dosimetric constraints for intestines were insufficient. That trial was attempted to reduce the volume of intestines receiving 35 Gy (V_35 Gy_), V_40 Gy_, and V_45 Gy_ [15], but more recent data suggest that the volume of intestines receiving low dose exposure, like V_15 Gy_ < 120 cc or 150 cc, may be important [16, 17]. We speculate that the reduction of the volume of intestines receiving irradiation, including low dose irradiation, would alleviate acute gastrointestinal toxicity in preoperative chemoradiotherapy for this population of patients.

The aim of the current trial is to explore how much irradiated volume was reduced by IMRT compared to 3DCRT in preoperative chemoradiotherapy for rectal cancer and to examine the safety and effectiveness of IMRT.

## MATERIALS AND METHODS

The Institutional Review Board of the Kyoto University Graduate School and Faculty of Medicine, Kyoto University Hospital approved the trial on 18 January 2017. The trial was registered with the University Hospital Medical Information Network Clinical Trials Registry (UMIN000024549; http://www.umin.ac.jp/ctr) and the Japan Registry of Clinical Trials (jRCTs051180039; http://www.jrct.niph.go.jp). Our institution had not used IMRT for preoperative chemoradiotherapy before the current study. It was arranged to conduct as a pilot trial. Twelve patients were at least recommended as the number of included patients in the pilot trial [18]. We estimated the number of the eligible patients was six to seven in one year. This trial planned to accrue 15 patients in 3 years.

### Eligibility criteria

Eligibility criteria were as follows: age 20–80 years; histologically confirmed rectal adenocarcinoma; primary locally advanced rectal tumor located below peritoneal reflection; T3–T4 and N0–N2 (Union for International Cancer Control, 7^th^ edition); no distant metastasis; Eastern Cooperative Oncology Group performance status 0–2; sufficient organ functions; and written consent to participate in the current trial. Sufficient organ functions were determined as follows: white blood cell count: 3000/mm^3^ < and ≤ 12 000/m^3^, neutrophil count ≥1500/mm^3^, platelet count ≥100 000/mm^3^, serum hemoglobin ≥9.0 g/dL, total bilirubin ≤1.5 mg/dL, both serum aspartate aminotransferase and serum alanine aminotransferase ≤100 IU/L and estimated creatinine clearance ≥60 ml/min. Colonoscopy, magnetic resonance imaging (MRI) of the pelvis and computed tomography (CT) of the neck, chest and abdomen were performed before enrollment.

Patients with the following characteristics were excluded from this study: active synchronous cancer; actively taking flucytosine, atazanavir sulfate or steroid medication; systemic infection; uncontrolled comorbidities such as diarrhea, ileus or interstitial pneumonitis, connective tissue disease, diabetes mellitus, hypertension, heart failure, pregnancy or breast-feeding for female patients and psychotic disorders.

### Clinical T- and N-staging

Clinical T-status was defined by digital examination and MRI [19, 20]. The circumferential resection margin (CRM) was judged as involved when the distance from primary rectal tumor or metastatic lymph nodes to mesorectal fascia or levator muscle was less than 1 mm or when the invasions to the intersphincteric plane were observed on MRI [21]. Peri-rectal lymph nodes with a short-axis diameter of more than 10 mm and lateral lymph nodes with a short-axis diameter of more than 7 mm were clinically diagnosed as metastatic lymph nodes.

### Radiotherapy planning

CT simulation was performed in a supine position with an individualized vacuum pillow or in prone position with a belly board. The primary rectal tumor and metastatic lymph nodes were contoured as gross tumor volumes using CT, MRI, colonoscopy and digital examination. The clinical target volumes of the primary rectal tumor (CTVprimary) and metastatic lymph nodes (CTVnode) were the volume of the primary rectal tumor, with a craniocaudal 2-cm margin along with rectum plus a cross-sectional 0.5-cm margin and the volume of metastatic lymph nodes plus a 0.5-cm margin, respectively. Subclinical lymph nodal regions included mesorectum, presacral, obturator and internal iliac lymph nodal regions. CTVprimary, CTVnode and subclinical lymph nodal regions with a 0.5–1.0-cm margin were defined as the planning target volume (PTV): PTVprimary, PTVnode and PTVsubclinical. Small and large intestine excluding rectum and bladder were defined as organs at risk. The delineation of intestines was based on the contouring guideline of Radiation Therapy Oncology Group [22]. Planning organ at risk volume for small intestines (PRV_Bowel_S) or large intestines (PRV_Bowel_L) was created as the volume of small intestines or large intestines plus a cross-sectional 0.3-cm margin, respectively. The intestines were delineated at least 2.0 cm above the most superior extent of the PTV. The peritoneal cavity excluding large intestines was also delineated, which was used by the previous IMRT trial to set dose constraints for small intestines [15].

**Fig. 1:**
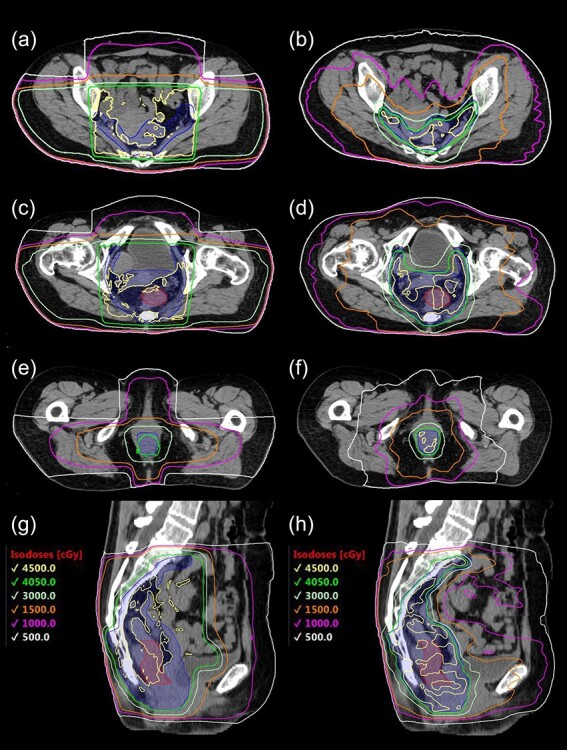
Axial and sagittal images of a representative dose distribution of 3DCRT (a, c, e, g) and IMRT in preoperative chemoradiotherapy (b, d, f, h) for rectal cancer. Red-filled contour, rectal primary tumor; Blue filled contour, PTV.

IMRT was generated by volumetric-modulated arc therapy (VMAT) using coplanar arcs. The dose was delivered to 50% of PTV, receiving 1.8 Gy per fraction. The current trial set two types of dose constraints for optimization: acceptable index and target index. The acceptable index was dose constraints mandatory to meet. The target index was dose constraints to meet if feasible. Target coverage was prioritized over sparing organs at risk in optimization. In each enrolled patient, VMAT plans were compared with 3DCRT plans which were created using the same CT images, for control, to explore how much volume of intestines and bladder were reduced by IMRT. 3DCRT plans were composed of the posterior–anterior field and lateral opposing fields including the PTV, with a 0.5 cm leaf margin, and doses were prescribed to the isocenter of each field. Representative dose distribution of 3DCRT and VMAT were illustrated in Fig. 1.

### Protocol preoperative chemoradiotherapy, evaluation and surgery

The treatment protocol comprised preoperative chemoradiotherapy and surgery. Doses were delivered by VMAT with 45 Gy in 25 fractions five days per week. Daily set-up errors were assessed and corrected using daily paired-kilovoltage images or on-board cone-beam CT. S-1 was orally administered on days 1 to 5, 8 to 12, 22 to 26 and 29 to 33, in a daily dose of 80 mg/body/day in patients with body surface area (BSA) < 1.25 m^2^; 100 mg/body/day in patients with BSA of 1.25–1.5 m^2^; and 120 mg/body/day in patients with BSA > 1.5 m^2^. Irinotecan was infused intravenously in a daily dose of 60 mg/m^2^ over 90 minutes on days 1, 8, 22 and 29. The dose of irinotecan was started from 40 mg/m^2^/day in patients with a genotype of uridine diphosphate glucuronosyltransferase1A1 ^*^6/^*^28, ^*^6/^*^6 or ^*^28/^*^28.

IMRT was interrupted during the presence of a neutrophil count <1000/mm^3^, platelet count <50 000/mm^3^, grade 3 diarrhea or febrile infection. S-1 and irinotecan were interrupted during the presence of the following: white blood cell count <3000/mm^3^, neutrophil count <1500/mm^3^, platelet count <100 000/mm^3^, serum total bilirubin >2.5 mg/dL, serum creatinine >1.5 mg/dL, serum aspartate aminotransferase or serum alanine aminotransferase >100 IU/L, grade 3 oral mucositis, grade 2 diarrhea or febrile infection.

S-1 and irinotecan were reduced in the next course when the following treatment-related adverse events had been observed in the previous course: neutrophil count <1000/mm^3^, platelet count <75 000/mm^3^, serum total bilirubin >2.5 mg/dL, serum creatinine >1.5 mg/dL, serum aspartate aminotransferase or serum alanine aminotransferase >100 IU/L, grade 3 oral mucositis or grade 2 diarrhea. The reduced dosages of S-1 in the next course were 50 mg/body/day in patients with BSA < 1.25 m^2^, 80 mg/body/day in patients with BSA of 1.25–1.5 m^2^, and 100 mg/body/day in patients with BSA > 1.5 m^2^. The reduced dosage of irinotecan in the next course was 40 mg/m^2^/day. The administration of S-1 or irinotecan were discontinued when the dosages of S-1 and irinotecan needed to be more reduced from the 50 mg/body/day and 40 mg/m^2^/day, respectively.

Clinical stage after preoperative chemoradiotherapy was evaluated using colonoscopy, MRI and CT from 6 weeks after the completion of preoperative chemoradiotherapy until the day of surgery. The disappearance of erosion or ulcer in the rectal lumen was judged as ycT0. The decrease of short-axis diameter less than 5 mm or disappearance of lymph nodal metastasis was diagnosed as ycN0. Patients underwent surgery until 10 weeks after completion of the preoperative chemoradiotherapy.

### Endpoints and statistical analyses

The primary endpoint was the number of patients with ≥ grade 2 acute gastrointestinal adverse events. Gastrointestinal adverse events included abdominal pain, anal pain, hemorrhoids, diarrhea, constipation, nausea, vomiting, mucositis, proctitis, fecal incontinence, rectal fistula, rectal stenosis, rectal bleeding and enteritis. These were evaluated at least once a week during preoperative chemoradiotherapy and every three weeks after completion of the preoperative chemoradiotherapy until the day of surgery, using the Common Terminology Criteria for Adverse Events, version 4.0. Adverse events within 90 days after the initial day of preoperative chemoradiotherapy were determined as acute. Adverse events, definitely, probably or possibly related to chemoradiotherapy occurring 91 days after the initial day of preoperative chemoradiotherapy, were defined as late.

The secondary endpoints were the numbers of late adverse events, dose reduction rate, downstaging rate, complete resection rate, clear pathological CRM rate, pathological complete response rate, perioperative complication evaluated by Clavien-Dindo classification [23], relapse-free survival (RFS) rate and overall survival (OS) rate. The dose reduction rate was defined as the proportion of patients who needed any dose reduction of chemotherapy or radiotherapy, to all enrolled patients. The downstaging rate was calculated as the proportion of clinically down-staged patients after preoperative chemoradiotherapy, to all enrolled patients. The rate of complete resection, clear pathological CRM and pathological complete remission were the proportion of patients who achieved complete resection, clear pathological CRM, and pathological complete remission to all patients who underwent surgery, respectively. Local regional control (LRC) was defined as the time from the day of enrollment to local-regional progression and censored on the date of no progression. RFS was defined as the time from the day of enrollment to disease progression and censored on the date of no progression. OS was defined as the time from the day of enrollment to death and censored on the date of the last visit. Rates of LRC, RFS and OS were calculated using the Kaplan–Meier method.

## RESULTS

### Patient cohort

From February 2017 to January 2020, 12 patients were enrolled (Table 1). The data of the current trial were fixed on March 18, 2021. The median follow-up period for the entire cohort was 32.5 months (range; 18–45). All primary tumors were located in the rectum close to or invading the anal canal. CRM was clinically involved in 11 patients.

**Table 1:** Patient characteristics (n = 12)

	(range)
Median age (range)	60 (38–69)
Gender (male/female)	4/8
Median body mass index (kg/cm^2^)	20.6 (16.6–24.9)
Eastern Cooperative Oncology Group performance status (0/1)	9/3
Uridine diphosphate glucuronosyltransferase1A1 genotype (^*^6/^*^28, ^*^6/^*^6 or ^*^28/^*^28/others)	2/10
Main tumor location (Rb or RbRa/RbP)	9/3
Median distance from anal verge (cm)	3 (0–6)
Circumferential resection margin (involved/not involved)	11/1
Clinical stage^†^	
T3/T4a/T4b	10/0/2
N0/N1/N2	2/9/1
Median value of carcinoembryonic antigen (ng/mL)	3.1 (1.6–17)

**Abbreviation:** Rb, rectum below peritoneal reflection; RbRa, Rb and rectum above peritoneal reflection; RbP, Rb and anal canal.

^†^Clinical stage was based on the Union for International Cancer Control 7^th^ edition.

### Feasibility of the protocol

**Fig. 2:**
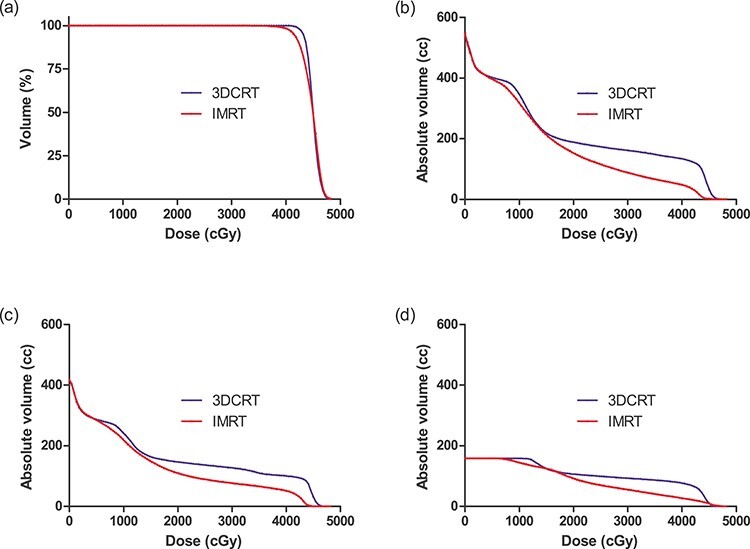
Mean dose-volume histograms of (a) PTV, (b) PRV Bowel_S, (c) PRV Bowel L, and (d) Bladder. Abbreviations: PRV_Bowel_S, the volume of small intestines plus a cross-sectional 0.3-cm margin; PRV_Bowel_L, volume of large intestines plus a cross-sectional 0.3-cm margin; 3DCRT, 3-dimensional conformal radiotherapy; IMRT, intensity-modulated radiotherapy.

All IMRT plans met the acceptable indices for target and organs at risk; however, the target dose index of V_15 Gy_ of small and large intestines were difficult to meet; this target index was met in only three and four patients, respectively (Table 2). Quantitative plan comparisons regarding mean dose-volume histograms in the enrolled patients showed that the volumes of intestine and bladder receiving the medium to high doses were reduced in IMRT compared to 3DCRT for control (Fig. 2).

**Table 2:** Dose constraints and actual dose

Structure	Dose-volume index	Dose constraints of intensity-modulated radiotherapy for the current protocol		Actual dose index		
		Target index (100% = 45 Gy)	Acceptable index (100% = 45 Gy)	Intensity-modulated radiotherapy for clinical use		Three-dimensional conformal radiotherapy for planning study
				Number of patients who met target/acceptable index (n = 12)	Mean value (range)	Mean value (range)
PTVprimary	D2%^¶^	<48.2 Gy (107%)	<51.7 Gy (115%)	12/0	47.3 Gy (46.7–48.0)	46.7 Gy (45.7–47.5)
	D98%^¶^	>42.7 Gy (95%)	>40.5 Gy (90%)	8/4	42.7 Gy (41.4–43.7)	42.4 Gy (41.2–43.5)
PTVnode	D2%^¶^	<48.2 Gy (107%)	<51.7 Gy (115%)	10/0^††^	47.3 Gy (46.0–47.9)	46.5 Gy (45.5–47.4)
	D98%^¶^	>42.7 Gy (95%)	>40.5 Gy (90%)	7/3^††^	42.8 Gy (40.8–44.6)	44.0 Gy (43.0–44.5)
PTV^*^	D2%^¶^	<49.5 Gy (110%)	<51.7 Gy (115%)	12/0	47.2 Gy (46.8–47.8)	47.1 Gy (46.3–47.6)
	D50%^¶^	45 Gy	-	12/0	45 Gy	45 Gy
	D98%^¶^	>40.5 Gy (90%)	>36 Gy (80%)	8/4	40.4 Gy (37.3–42.1)	42.5 Gy (41.3–43.0)
PTV-PRVs^†^	D2%^¶^	<48.2 Gy (107%)	<49.5 Gy (110%)	12/0	47.3 Gy (46.8–47.8)	47.1 Gy (46.3–47.7)
	D98%^¶^	>42.7 Gy (95%)	>38.2 Gy (85%)	12/0	40.8 Gy (38.3–42.6)	42.4 Gy (41.2–43.0)
Overlap PTV_PRV^‡^	D2%^¶^	<45 Gy (100%)	<51.7 Gy (115%)	11/1	44.4 Gy (43.8–46.4)	46.3 Gy (45.4–47.4)
	D98%^¶^	>40.5 Gy (90%)	> 36 Gy (80%)	5/7	39.3 Gy (36.2–41.0)	43.3 Gy (42.3–44.1)
PRV_Bowel_S^§^	V_15 Gy_^*^^*^	<120 cc	None	3/9	212 cc (20–660)	218 cc (16–539)
	V_45 Gy_^*^^*^	≤0 cc	None	10/2	1.3 cc (0–14)	28 cc (0–117)
PRV_Bowel_L^||^	V_15 Gy_^*^^*^	<120 cc	None	4/8	149 cc (49–278)	165 cc (69–316)
	V_45 Gy_^*^^*^	≤0 cc	None	10/2	0.4 cc (0–3)	32 cc (9–93)
Peritoneal cavity excluding large intestines	V_15 Gy_^*^^*^	None	None	Not available	271.925 cc (69.8–822)	268.3 cc (64.6–650.2)
	V_35 Gy_^*^^*^	None	None	Not available	84.6 cc (1.8–351.1)	178.8 cc (1.7–489)
	V_40 Gy_^*^^*^	None	None	Not available	60.1 cc (0.8–246.3)	160.9 cc (0.7–456)
	V_45 Gy_^*^^*^	None	None	Not available	6.9 cc (0–23.5)	38.2 cc (0.2–142.6)

**Abbreviations:** PTV, planning target volume; PTVprimary, PTV for primary rectal tumor; PTVnode, PTV for metastatic lymph nodes. PRV, planning organ at risk volume.

^*^PTV, the volume of PTVprimary plus PTVnode.

^†^PRVs, the volume of PRV_Bowel_S and PRV_Bowel_L.

^‡^OverlapPTV_PRV, the overlapping volume of PTV and PRVs.

^§^PRV_Bowel_S, the volume of small intestines plus a cross-sectional 0.3-cm margin.

^||^PRV_Bowel_L, volume of large intestines plus a cross-sectional 0.3-cm margin.

^¶^D_X%_, dose covering X% of the volume.

^*^^*^V_X Gy_, the volume receiving X Gy.

^††^no lymph nodal metastasis in two patients.

**Fig. 3:**
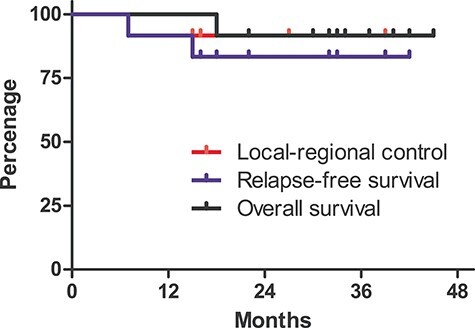
Kaplan–Meier curves for LRC, RFS and OS.

All enrolled patients finished the chemoradiotherapy protocol; however, two patients refused to undergo surgery after preoperative chemoradiotherapy. Planned radiotherapy sessions were completed in all enrolled patients, with a median overall treatment time of 37 days (range: 36–49). The chemotherapy dose was reduced in four patients, due to grade 3 neutropenia in three patients and grade 2 diarrhea in one patient. The dose reduction rate was 33%.

### Acute and late adverse events and perioperative complications

Grade 4 hematologic adverse events were not observed. Anorectal symptoms were the most common ≥ grade 2 non-hematologic adverse events and they were observed in three patients already before the treatment protocol (Table 3). Excluding anorectal symptoms, two patients (17%) experienced ≥ grade 2 acute gastrointestinal adverse events related to the treatment protocol (Table 4). Three patients who met V_15 Gy_ of PRV_Bowel_S < 120 cc were free of ≥ grade 2 acute gastrointestinal adverse events, excluding anal pain.

**Table 3:** Details of acute adverse events (n = 12)

	Grade 0–1	Grade 2	Grade 3
Hematologic			
Leukocytopenia	8	0	4
Neutropenia	7	1	4
Anemia	10	1	1
Thrombocytopenia	12	0	0
Non-hematologic			
Gastrointestinal			
Abdominal pain	10	1	1
Anal pain	8	3^*^	1^†^
Diarrhea	11	1	0
Constipation	11	1	0
Nausea/Vomiting	12	0	0
Mucositis	12	0	0
Proctitis	12	0	0
Fecal incontinence	12	0	0
Rectal fistula	12	0	0
Rectal stenosis	10	2^‡^	0
Enterocolitis	11	0	1
Bleeding	12	0	0
Non-gastrointestinal			
Fever	12	0	0
Fatigue	10	2	0
Body weight loss	10	2	0
Radiationdermatitis	12	0	0
Appetite loss	10	2	0
Urinary frequency/incontinence	12	0	0
Infection	12	0	0

^*^Adverse event existed before treatment protocol in two patients.

^†^Adverse event existed before treatment protocol in one patient.

^‡^Adverse event existed before treatment protocol in two patients.

**Table 4:** Response and adverse events in 12 patients

	UGT1A1	Before preoperative chemoradiotherapy			≥Grade 2 acute gastrointestinal adverse event^‡^	After preoperative chemoradiotherapy		Operation	Surgical specimen			≥Grade II perioperative comorbidity^§^	≥Grade 2 late adverse event^††^	Disease progression or relapse
		cStage^†^	clinical CRM	Distance from anal verge (cm)		ycStage^†^	ycCRM		ypStage^†^	R0 resection	ypCRM			
1	^*^1/^*^6	T3N1	Involved	4	None	T0N1	Involved	Refused	−	−	−	−	None	Local progression
2	^*^1/^*^1	T3N0	Involved	0	Grade 3 anal pain^||^ and enterocolitis. Grade 2 general fatigue, anorexia, abdominal pain, diarreha, and constipation.	T3N0	Involved	Laparoscopy-assisted abdominoperineal resection	T2N0	Done	≤1 mm	None	Grade 3 perineal hernia	No relapse
3	^*^1/^*^1	T3N1	Involved	2	Grade 2 anal pain	T2N1	Not involved	Robot-assisted ISR	T0N1	Done	1 mm<	None	None	No relapse
4	^*^1/^*^1	T4bN1	Involved	4	Grade 2 anal pain^||^ and rectal stenosis^||^	T3N0	Not involved	Robot-assisted ISR	T3N0	Done	1 mm<	None	Grade 3 cellulitis, and Grade 2 anastomotic stenosis	No relapse
5	^*^1/^*^6	T3N1	Involved	6	None	T3N0	Not involved	Robot-assisted LAR	T3N0	Done	1 mm<	Grade II hypoglycemia	None	No relapse
6	^*^1/^*^1	T3N1	Involved	4	Grade 2 rectal stenosis^||^	T3N0	Not involved	Robot-assisted LAR	TisN0	Done	1 mm<	Grade II obturator neuropathy	None	Pulmonary metastasis
7	^*^1/^*^28	T4bN1	Involved	2	None	T3N1	Not involved	Laparoscopy-assisted-ISR	T3N1	Done	≤1 mm	None	None	No relapse
8	^*^1/^*^6	T3N1	Involved	2	Grade 2 anal pain	T3N0	Not involved	Robot-assisted ISR	T3N0	Done	1 mm<	None	None	No relapse
9	^*^6/^*^6	T3N1	Not involved	2	Grade 2 abdominal pain	T2N0	Not involved	Robot-assisted ISR	T1N1	Done	1 mm<	Grade II chylous ascites	None	No relapse
10	^*^1/^*^1	T3N1	Involved	2	None	T0N0	Not involved	Refused	−	−	−	−	None	No progression
11	^*^1/^*^1	T3N0	Involved	5	None	T3N0	Not involved	Robot-assisted LAR	T3N0	Done	1 mm<	None	None	No relapse
12	^*^6/^*^28	T3N2	Involved	5	None	T3N2	Involved	Robot-assisted LAR	T3N2b	Done	1 mm<	None	None	No relapse

**Abbreviations:** UGT, uridine diphosphate-glucuronosyltransferase; CRM, circumferential resection margin, ISR, intersphincteric resection LAR, low anterior resection.

^†^Clinical and pathological stage was based on the criteria of the seventh edition of the Union for International Cancer Control TNM staging system.

^‡^Adverse events were evaluated according to Common Terminology Criteria for Adverse Events, version 4.0.

^§^Perioperative comorbidity was scored by Clavian-Dindo classification.

^||^Observed toxicities before treatment protocol.

Grade 2 perioperative comorbidities were observed in three of the 10 patients who underwent surgery (Table 4). More than grade 2 late adverse events were observed in two patients; grade 3 perineal hernia in one patient after 16 months; and grade 3 cellulitis after 7 months and grade 2 anastomotic stenosis after 11 months in the other patient (Table 4).

### Effectiveness

The clinical downstaging rate was 75% (9/12) in the intention-to-treat cohort. Anal sphincter preserving surgery with temporary ileostomy was performed in 90% (9/10) of the patients who underwent surgery. Lateral lymph nodal dissection was performed when lateral lymph nodal metastasis was suspected before preoperative chemoradiotherapy. The rate of complete resection and clear pathological CRM in patients who underwent surgery was 100% (10/10) and 80% (8/10), respectively. Pathological complete response of primary rectal tumor was observed in one patient (Table 4).

Two patients experienced disease progression during follow-up: mesorectal lymph nodal progression 7 months after the enrollment in one patient who refused surgery; and multiple pulmonary metastasis 15 months after the enrollment in one patient. The 3-year LRC rate, RFS rate and OS rate were 92% (95% condence interval [CI], 54–99%), 83% (95% CI, 48–96%) and 92% (95% CI, 54–99%), respectively (Figure 3).

## DISCUSSION

The current IMRT protocol reduced the volume of intestines receiving medium to high doses while delivering adequate doses to target, compared to 3DCRT. This might alleviate severe acute gastrointestinal adverse events related to irradiation of the intestines; however, reduction of the volume of intestine receiving low dose was not always feasible. High LRC rate and sphincter preservation suggested that the effectiveness of preoperative chemoradiotherapy was not compromised by the current IMRT protocol.

The adequate volume reduction of small intestines receiving medium to high doses might reduce acute gastrointestinal toxicity. The current trial showed that IMRT reduced the volume of small intestines receiving medium to high doses compared with those of 3DCRT. The current IMRT trial additionally achieved the larger volume reduction of small intestines receiving medium to high doses compared with the previous IMRT trial: the median value of V_35 Gy_, V_40 Gy_ and V_45 Gy_ of the peritoneal cavity excluding large intestines was the half of the values of dose constraints set by the multi-institutional phase II trial: V_35 Gy_ < 180 cc, V_40 Gy_ < 100 cc and V_45 Gy_ < 65 cc [15]. This large adequate reduction might contribute to the decrease of the acute gastrointestinal toxicity in the current trial while 52% and 18% of patients experienced more than grade 2 and grade 3–4 acute gastrointestinal toxicity in the previous phase II trial of IMRT for rectal cancer [15]. The adequate reduction of the medium to high doses of small intestines by IMRT might alleviate the acute gastrointestinal toxicity which was not achieved by the previous IMRT trial. The merit of the reduction of medium to high doses needs to be further explored as well as the reduction of the low dose exposure to small intestines.

Consistent with the previous studies [16, 17], the patients with V_15 Gy_ of PRV_Bowel_S < 120 cc were free of ≥ grade 2 acute gastrointestinal adverse events excluding from anal pain in the current trial. The low dose irradiated volume of the small intestines was considered to be associated with acute gastrointestinal toxicity; however differently from medium to high dose exposure, the current trial showed that the reduction of the low dose irradiated volume of intestines was not always feasible. Low dose exposures to normal tissue surrounding the PTV were reported to be difficult to reduce, even using IMRT [24]. The volume of visceral fat and the anatomical location of small intestines to the PTV were anticipated as a key to meet the dose constraints of small intestines; however the current study did not obtain any definite characteristics. Recently, more elaborated radiotherapy planning using avoidance structure and sector were reported to enable VMAT to reduce V_15 Gy_ of intestines in the planning study [25]. Further studies are needed to ensure the reduction of irradiated volume of intestines, especially for low dose exposure, in preoperative chemoradiotherapy using IMRT for rectal cancer.

In future trials, a specified endpoint is necessary to evaluate the clinical advantages of IMRT for rectal cancer. Irradiation fields for rectal cancer always include the anorectum, irrespective of 3DCRT or IMRT, which likely causes anorectal toxicity. In addition, low rectal cancer is located close to the anus. The invasion of rectal cancer to the anus itself likely causes anal pain. The current trial showed that anorectal symptoms were the most common ≥ grade 2 acute gastrointestinal adverse events. Similarly, a previous phase II trial showed high incidence of anorectal symptoms after preoperative chemoradiotherapy for rectal cancer [11, 26]. The only one phase II trial which failed to show the reduction of gastrointestinal acute toxicity by IMRT, defined the primary endpoint as preoperative treatment-related gastrointestinal adverse events [15]. It did not exclude anorectal symptoms, which might be associated with the negative result [15]. Anus- or rectum-related symptoms should be excluded from gastrointestinal toxicities when exploring the clinical advantages of IMRT in preoperative chemoradiotherapy for rectal cancer. A clinical trial with a specified endpoint potentially helps to confirm the clinical advantage of IMRT in preoperative chemoradiotherapy for rectal cancer.

The current study exhibited some limitations. A selection bias was present due to the small number of selected patients, without a control group. The evaluation of adverse events by non-blinded observers was largely affected by a detection bias. Also, an attrition bias was present due to refusal of surgery, and an early termination bias was present due to poor accrual. Finally the structure to estimate the irradiated volumes of small intestines in the current study was not identical to that of the previous IMRT trial [15]. The current study used the structure of PRV_Bowel_S to set dose constraints for small intestines; however the previous IMRT trial used the structure of the peritoneal space excluding large intestines. The issue of comparability between the previous IMRT trial and the current one existed. Further trials minimizing the above bias and problems are necessary to gain the rationales of IMRT in preoperative chemoradiotherapy for rectal cancer.

The use of IMRT for rectal cancer already increased from 1% in 2004 to 22% in 2014 in the USA [27]. In a survey of radiotherapy centers in the UK, 68% of responders used IMRT for all patients with rectal cancer [28], despite the lack of clinical evidence of using IMRT for rectal cancer. The data of the clinical outcomes with detailed dose-volume metrics of the current IMRT trial may help physicians to adopt IMRT planning in preoperative chemoradiotherapy for rectal cancer. In conclusion, preoperative chemoradiotherapy using the current IMRT protocol for rectal cancer potentially achieved high LRC and minimal acute gastrointestinal toxicity, although further advancement is required to reduce the irradiated volume of intestines, especially those receiving low doses.

## CLINICAL TRIAL NUMBER

UMIN000024549 and jRCTs051180039.
